# 3T MRI investigation of cardiac left ventricular structure and function in a UK population: The tayside screening for the prevention of cardiac events (TASCFORCE) study

**DOI:** 10.1002/jmri.25267

**Published:** 2016-05-03

**Authors:** Stephen J. Gandy, Matthew Lambert, Jill Belch, Ian Cavin, Elena Crowe, Roberta Littleford, Jennifer A. MacFarlane, Shona Z. Matthew, Patricia Martin, R. Stephen Nicholas, Allan Struthers, Frank Sullivan, Shelley A. Waugh, Richard D. White, Jonathan R. Weir‐McCall, J. Graeme Houston

**Affiliations:** ^1^ NHS Tayside Clinical RadiologyNinewells HospitalDundeeUK; ^2^ NHS Tayside Medical PhysicsNinewells HospitalDundeeUK; ^3^ University of Dundee School of MedicineDundeeUK; ^4^ Department of Family and Community MedicineUniversity of TorontoTorontoCanada; ^5^ Department of Research and InnovationNorth York General HospitalTorontoCanada; ^6^ Department of Clinical RadiologyUniversity Hospital of WalesUK

**Keywords:** cardiac, MRI, 3.0T, left ventricle, population

## Abstract

**Purpose:**

To scan a volunteer population using 3.0T magnetic resonance imaging (MRI). MRI of the left ventricular (LV) structure and function in healthy volunteers has been reported extensively at 1.5T.

**Materials and Methods:**

A population of 1528 volunteers was scanned. A standardized approach was taken to acquire steady‐state free precession (SSFP) LV data in the short‐axis plane, and images were quantified using commercial software. Six observers undertook the segmentation analysis.

**Results:**

Mean values (±standard deviation, SD) were: ejection fraction (EF) = 69 ± 6%, end diastolic volume index (EDVI) = 71 ± 13 ml/m^2^, end systolic volume index (ESVI) = 22 ± 7 ml/m^2^, stroke volume index (SVI) = 49 ± 8 ml/m^2^, and LV mass index (LVMI) = 55 ± 12 g/m^2^. The mean EF was slightly larger for females (69%) than for males (68%), but all other variables were smaller for females (EDVI 68v77 ml/m^2^, ESVI 21v25 ml/m^2^, SVI 46v52 ml/m^2^, LVMI 49v64 g/m^2^, all *P* < 0.05). The mean LV volume data mostly decreased with each age decade (EDVI males: –2.9 ± 1.3 ml/m^2^, females: –3.1 ± 0.8 ml/m^2^; ESVI males: –1.3 ± 0.7 ml/m^2^, females: –1.7 ± 0.5 ml/m^2^; SVI males: –1.7 ± 0.9 ml/m^2^, females: –1.4 ± 0.6 ml/m^2^; LVMI males: –1.6 ± 1.1 g/m^2^, females: –0.2 ± 0.6 g/m^2^) but the mean EF was virtually stable in males (0.6 ± 0.6%) and rose slightly in females (1.2 ± 0.5%) with age.

**Conclusion:**

LV reference ranges are provided in this population‐based MR study at 3.0T. The variables are similar to those described at 1.5T, including variations with age and gender. These data may help to support future population‐based MR research studies that involve the use of 3.0T MRI scanners. J. Magn. Reson. Imaging 2016;44:1186–1196.

The use of cardiac magnetic resonance (MR) for the assessment of left ventricular (LV) structure and function is a well‐established technique that is used for both clinical and research investigations. Volunteer “normal ranges” have been published for data acquired on 1.5T systems,^1^, ^2^, ^3^ with an emphasis on the use of the steady state‐gradient echo sequence since the associated *T*_2_/*T*_1_ weighting provides excellent contrast between the myocardium and the blood pool. Other research groups have extended this work to acquire MR LV data on larger‐scale populations. Examples of these include the following studies: Framingham Offspring,^4^ Dallas Heart,^5^ MESA,^6^ AGES Reykjavik,^7^ German SHIP,^8^ and Lichfield LARGE Heart.^9^ Further large‐scale investigations such as the UK Biobank^10^ are also in progress. However, to date all population‐based cardiac MR studies have been conducted using 1.5T scanners.

With the increasing use of 3.0T MR scanners for clinical imaging, there is a need to establish equivalent data for images acquired at this higher field strength.^11^ Small comparison studies of 1.5T vs. 3.0T MR in healthy volunteers have previously been undertaken.^12^, ^13^ Recent work from Liu et al has reported the use of a 3.0T MR system for MR scanning of a population of healthy African Americans,^14^ although it is known from previous work that ethnic variations in LV structure and function do exist,^15^ so there might be a need to extend this work to a European setting.

Early MR investigations that used 3.0T machines tended to recommend the use of spoiled gradient echo imaging since it was less susceptible to flow‐related artifacts.^16^ However, with the development over time of better shimming techniques, the steady‐state sequence has become the sequence of choice for MR at 3.0T.^17^

The *T*_1_ and *T*_2_ relaxation times of tissues are inherently affected by the local magnetic field strength to which they are exposed, and *T*_1_ relaxation times in particular are elevated at higher field strengths.^18^ In cardiac MR, since the process of computer segmentation involves the precise delineation of myocardial boundaries, it therefore follows that boundary delineation could be perceived differently at 1.5T and 3.0T due to possible variations in myocardium‐blood contrast‐to‐noise ratio (CNR).^19^ Such systematic differences could be clinically important in cases where particular LV “cutoff” values are used to determine the future course of patient treatments; or in longitudinal investigations where comparisons may involve datasets acquired on machines of different field strengths. From a clinical perspective, since LV hypertrophy is an indicator of many underlying cardiac conditions and can also be a strong independent predictor for incident cardiovascular events, a precise definition of population‐based ranges is required.^20^

The objective of this study therefore was to use MR to examine the LV structure and function of a large UK population of volunteers using a standard steady‐state gradient echo sequence on a 3.0T scanner in order to establish population range data capable of comparison with similar data acquired at 1.5T.

## Materials and Methods

This MR study was conducted as part of a wider population‐based cardiovascular MR investigation of volunteers asymptomatic of cardiovascular disease (CVD) (the Tayside Screening for the Prevention of Cardiac Events [TASCFORCE] study). The study was allocated an International Standard Randomised Control Trial Number: ISRCTN38976321. Local research ethical committee (REC) approval for the work was obtained and all volunteers gave informed consent. A total of 1528 volunteers were included in the study, which ran from June 2008 until February 2013. Inclusion criteria were as follows: 1) age ≥40 years; 2) free from CVD or other indication for statin therapy as recommended by the Scottish Intercollegiate Guidelines Network (SIGN) report 97 (www.sign.ac.uk) published in February 2007; 3) 10‐year risk of coronary heart disease below 20% as predicted by the Adult Treatment Panel III (ATPIII) algorithm^21^; and 4) a plasma B type natriuretic peptide (BNP) level greater than the gender specific median. Exclusion criteria included: i) pregnancy; ii) known primary muscle disease; iii) known atherosclerotic disease, including unstable angina, previous myocardial infarction, peripheral arterial disease, amputation, revascularization, hypertension, heart failure, or cerebrovascular event; iv) known diabetes; v) active liver disease; vi) other known illness or contraindication to MR; vii) participation in a clinical trial; viii) inability to give informed consent; ix) known alcohol abuse; and x) blood pressure (BP) of greater than 145/95 mmHg.

Each volunteer was grouped into a “decade band” based on their age at the time of the investigation. The decade bands were defined by age as follows: 1) the “40s” (40–49 years); 2) the “50s” (50–59 years); 3) the “60s” (60–69 years); and 4) the “over 70s” (≥70 years).

The MR protocol has been described in detail elsewhere,^22^ but in brief imaging was performed in the head‐first supine orientation using a 3T [102x32] Scanner (Magnetom Trio, Siemens, Erlangen, Germany). A body matrix radiofrequency (RF) coil (six elements) was used in combination with a spine array (up to 24 elements).

Three plane localizer steady‐state gradient echo images of the heart were initially obtained using a true fast imaging with steady‐state free precession (TrueFISP) sequence, and these were followed by the acquisition of further localizers in the cardiac two‐chamber (2ch), four‐chamber (4ch), and short axis (SA) orientations. Subsequently, electrocardiogram (ECG)‐gated segmented breath‐hold cinematic (CINE) TrueFISP images were acquired in the LV 4ch and 2ch orientations, and a stack of SA images were acquired from the atrio‐ventricular ring to the LV apex using 2D ECG‐gated breath‐hold segmented CINE TrueFISP sequence with retrospective gating. The sequence parameters for the short‐axis acquisitions were: TR/TE = 3.4/1.5 msec, flip angle >50°, field of view >360 mm (volunteer dependent), pixel matrix 173 × 256, slice thickness 6 mm and interslice gap 4 mm (slices acquired every 10 mm). Retrospective ECG gating was used, with 25 cardiac phases reconstructed (25 lines per segment) and two image slices acquired per breath‐hold. Parallel imaging was also implemented (integrated parallel acquisition technique, iPAT x2), resulting in a scan time of <15 seconds per breath‐hold.

### Image Analysis

All datasets were analyzed once by one member of a team of six medical physics observers (S.G., R.N., J.M., S.W., S.M., and I.C., cardiac MR experience ranging from 7 to 12 years). This was performed on a rotational basis in order to ensure (as far as possible) that each observer was responsible for segmenting an equal number of datasets. The images were analyzed using Argus (Siemens Multi‐modality Work Platform, VB15 and VB17). Region of interest (ROI) contours were placed around endocardial and epicardial LV borders on all image slices at end‐diastole and end‐systole that contained 50% or more full‐thickness myocardium. Quantitative measurements of ejection fraction (EF), end‐diastolic volume (EDV), end‐systolic volume (ESV), stroke volume (SV), and LV mass (LVM) (at end‐diastole) were derived. Papillary muscles were included in the LVM if visually indistinguishable from the myocardial wall, but otherwise assigned to the left ventricular blood pool. As far as possible (within the constraints of the software capability) the adopted methodology was performed as per the guidance notes provided by Schulz‐Menger et al.^23^

### Statistical Analysis

All original study participants were included in the baseline analysis, as a representative UK population of individuals asymptomatic of CVD. A subanalysis of the full cohort was also performed in order to identify those volunteers (“subset cohort”; *n* = 782) who, in addition to being asymptomatic, had more stringent low‐risk factor criteria for future CVD. Participants were assigned to this subset if they had BP < 140/90 mmHg and no history of smoking,^6^ together with plasma BNP lower than 2 SDs above the full cohort gender‐specific mean (30.60 pg/ml for men and 53.36 pg/ml for women).

Normalization of MR LV data to body surface area (BSA) was performed using the simple formula described by Mosteller.^24^ Data were presented as mean ± SD in all cases.

Comparison of the mean values of each LV parameter between male and female cohorts was performed using a Student's *t*‐test, with *P* < 0.05 indicating significant differences between the two genders. The association of all LV parameters with age (for each gender) was evaluated, and a one‐way analysis of variance (ANOVA) (with Tukey post‐hoc analysis) was performed (four samples per gender) in order to identify statistical differences between the mean values of each of these variables with age. Regression analysis was also performed to investigate the various associations between each LV variable and age. From this, a “per decade” change in each variable was calculated, based on the assumption that the changes were linear with age. A formal assessment of intra‐ and interobserver repeatability has not been presented since this is reported elsewhere.^22^ However, reasonable estimates of interobserver variation can be extracted from the data generated by different segmentation observers because each of the respective cohorts were age‐matched, gender‐matched, and normalized to BSA. Comparison of 3T data versus 1.5T data was performed by tabulating the pooled data reported by Kawel‐Boehm et al^11^ against the data acquired in this study. Data were presented for both field strengths as a mean ± SD for all LV variables, and stratified according to gender. Full data ranges for each LV variable were also recorded. Differences between the means of each variable were calculated by simple subtraction in order to estimate whether the LV variable means varied randomly between 1.5T and 3.0T, or whether the means were systematically different. All statistical testing was performed using SPSS (IBM, Armonk, NY).

## Results

A total of 1515 volunteers were scanned successfully, and a description of demographic information related to anatomic size is shown in Table 1. A further *n* = 13 volunteers were also scanned (original study size *n* = 1528) but were excluded from the analysis as a result of either radiographic error or significant movement artifacts experienced during the MR scanning process.

**Table 1:** Demographic Information Related to Anatomical Size for All Volunteers in the Study

ABSOLUTE	No Volunteers	Height (m)	Weight (kg)	BMI (kg/m^2^)	BSA (Mosteller)	BSA (DuBois)
All	1515 (100%)	1.69 (0.09)	75.04 (14.31)	26.18 (4.23)	1.87 (0.21)	1.85 (0.20)
Males	574 (37.9%)	1.77 (0.07)	83.53 (12.28)	26.52 (3.50)	2.02 (0.17)	2.01 (0.16)
Females	941 (62.1%)	1.64 (0.07)	70.54 (13.00)	26.25 (4.44)	1.79 (0.18)	1.76 (0.16)
						
Males (40s)	197 (13.0%)	1.79 (0.07)	85.02 (12.65)	26.59 (3.53)	2.05 (0.17)	2.03 (0.16)
Males (50s)	235 (15.5%)	1.77 (0.07)	84.11 (12.13)	26.72 (3.41)	2.03 (0.17)	2.01 (0.15)
Males (60s)	118 (7.7%)	1.76 (0.06)	80.68 (11.61)	26.13 (3.46)	1.98 (0.16)	1.96 (0.15)
Males (≥70s)	24 (1.6%)	1.76 (0.06)	79.72 (11.41)	25.95 (4.27)	1.97 (0.15)	1.95 (0.13)
						
Females (40s)	318 (21.0%)	1.65 (0.07)	72.09 (14.33)	26.48 (4.98)	1.81 (0.19)	1.79 (0.17)
Females (50s)	371 (24.5%)	1.64 (0.07)	70.43 (12.64)	26.22 (4.65)	1.78 (0.17)	1.76 (0.15)
Females (60s)	213 (14.1%)	1.63 (0.06)	66.50 (10.71)	25.05 (3.95)	1.73 (0.15)	1.71 (0.14)
Females (≥70s)	39 (2.6%)	1.62 (0.07)	64.61 (9.55)	24.56 (3.55)	1.70 (0.14)	1.69 (0.13)

Data are stratified by gender and age, and other parameters such as mean body mass index (BMI) and mean body surface area (BSA) are included. The mean BSA was calculated using the formulae described by Mosteller^24^ (BSA Mosteller) and DuBois‐DuBois^29^ (BSA DuBois). The values for height, weight, BMI and BSA are represented as means ‐ with standard deviations alongside in parentheses.

A summary of the results is shown in Table 2. When the normalized data from all female and male participants were examined together the mean EF was higher for females (*P* < 0.05), but all other variables (EDV, ESV, SV, and LVM) were higher for males (all *P* < 0.05).

**Table 2:** LV Structure and Function Data Acquired on a Cohort of 1515 Volunteers

ABSOLUTE	No Volunteers	EF (%)	EDV (ml)	ESV (ml)	SV (ml)	LVM (g)
All	1515 (100%)	69 ± 6	133 ± 29	42 ± 15	91 ± 19	103 ± 29
Males	574 (37.9%)	68 ± 6	155 ± 28	50 ± 15	105 ± 19	129 ± 24
Females	941 (62.1%)	69 ± 7	120 ± 21	37 ± 12	82 ± 14	87 ± 17
						
Males (40s)	197 (13.0%)	67 ± 6	163 ± 27	54 ± 13	109 ± 20	135 ± 27
Males (50s)	235 (15.5%)	68 ± 6	153 ± 27	49 ± 15	104 ± 18	128 ± 22
Males (60s)	118 (7.7%)	68 ± 7	147 ± 26	47 ± 15	100 ± 17	123 ± 21
Males (≥70s)	24 (1.6%)	68 ± 6	143 ± 32	47 ± 15	97 ± 21	122 ± 24
						
Females (40s)	318 (21.0%)	68 ± 6	127 ± 20	41 ± 11	86 ± 14	88 ± 17
Females (50s)	371 (24.5%)	69 ± 7	121 ± 21	38 ± 12	83 ± 14	88 ± 17
Females (60s)	213 (14.1%)	71 ± 7	110 ± 19	33 ± 12	78 ± 12	84 ± 16
Females (≥70s)	39 (2.6%)	72 ± 6	104 ± 18	30 ± 10	74 ± 12	81 ± 15

**NORMALISED**	No Volunteers	EF (%)	EDVI (ml/m^2^)	ESVI (ml/m^2^)	SVI (ml/m^2^)	LVMI (g/m^2^)
All	1515 (100%)	69 ± 6	71 ± 13	22 ± 7	49 ± 8	55 ± 12
Males	574 (37.9%)	68 ± 6	77 ± 13	25 ± 7	52 ± 9	64 ± 10
Females	941 (62.1%)	69 ± 7	68 ± 11	21 ± 7	46 ± 7	49 ± 8
						
Males (40s)	197 (13.0%)	67 ± 6	80 ± 13	26 ± 7	53 ± 9	66 ± 12
Males (50s)	235 (15.5%)	68 ± 6	76 ± 13	24 ± 8	52 ± 9	63 ± 10
Males (60s)	118 (7.7%)	68 ± 7	74 ± 13	24 ± 8	51 ± 9	62 ± 10
Males (≥70s)	24 (1.6%)	68 ± 6	73 ± 14	24 ± 7	49 ± 9	62 ± 10
						
Females (40s)	318 (21.0%)	68 ± 6	70 ± 10	22 ± 6	48 ± 7	49 ± 8
Females (50s)	371 (24.5%)	69 ± 7	68 ± 11	21 ± 7	47 ± 7	49 ± 8
Females (60s)	213 (14.1%)	71 ± 7	64 ± 10	19 ± 7	45 ± 7	49 ± 8
Females (≥70s)	39 (2.6%)	72 ± 6	61 ± 9	18 ± 5	43 ± 6	48 ± 8

The presented data (mean ± SD) are stratified by gender and also by age decades. Normalization of the absolute values to body surface area was performed using the Mosteller formula. EF = ejection fraction, EDV = end diastolic volume, ESV = end systolic volume, SV = stroke volume, LVM = left ventricular mass, EDVI = end diastolic volume index, ESVI = end systolic volume index, SVI = stroke volume index, LVMI = left ventricular mass index. Statistically significant differences were detected for all mean LV variables between the male (*n* = 574) and female (*n* = 941) cohorts (*P* < 0.05).

**Figure 1:**
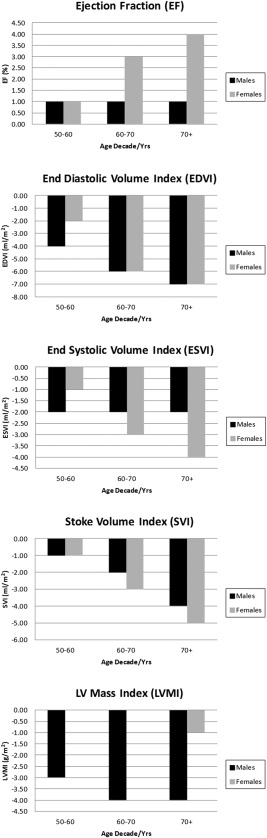
A plot of mean ejection fraction (EF), end‐diastolic volume (EDV), end‐systolic volume (ESV), stroke volume (SV), and left ventricular mass (LVM) showing the change of each variable with age in males and females for cohorts in the age ranges 50–59 years (50s), 60–69 years (60s) and over 70 years (≥70s), relative to the baseline 40–49 years (40s) cohort.

When the LV variables were subdivided into age decade categories (40s, 50s, 60s, and ≥70s) a number of age‐related changes were evident. For males, the mean EF rose slightly over the first decade studied (+1%, *P* < 0.04) but then remained stable across the remaining age decades. However, the means of all other variables were reduced with age (Fig. 1) (40s vs. ≥70s; EDVI and SVI *P* < 0.05).

For females, the mean EF rose marginally with age (a 4.0% increase was noted in the ≥70 years cohort relative to the 40–49 years cohort) but the means of all other volumetric variables were noted to decrease with age (40s vs. ≥70s; EDVI, ESVI, SVI *P* < 0.05). The most stable of all the variables was found to be the mean LVMI, which was only reduced by 1 g/m^2^ in the ≥70 years cohort relative to the 40s cohort. This pattern appeared different from that observed in the male cohort, which demonstrated a more defined reduction in mean LVMI with age.

The results of regression analysis describing the linear change of each LV variable per decade of age are highlighted in Table 3. All variables except for EF were noted to decrease with age, and the EDVI showed the biggest “per decade” reduction (–2.9 ± 1.3 ml/m^2^ for males and –3.1 ± 0.8 ml/m^2^ for females). The biggest difference between the male and female population was noted for LVMI, where greater “per decade” losses were measured in the male cohort (–1.6 ± 1.1 g/m^2^) relative to the female cohort, which remained virtually stable (–0.2 ± 0.6 g/m^2^).

**Table 3:** Results of Linear Regression Analysis Performed on the Male and Female Cohorts in Order to Derive “Per Decade” Change for Each of the Measured Indexed LV Variables

	Correlation Coefficient r (95% CI)	Slope (95% CI)	y‐intercept	Per Decade Change (95% CI)
**Male Age (x‐variable)**				
EF (%)	0.08	0.06	64.66	0.6 (±0.6)
EDVI (ml/m^2^)	−0.18	−0.29	92.67	−2.9 (±1.3)
ESVI (ml/m^2^)	−0.14	−0.13	31.81	−1.3 (±0.7)
SVI (ml/m^2^)	−0.15	−0.17	60.86	−1.7 (±0.9)
LVMI (g/m^2^)	−0.13	−0.16	72.69	−1.6 (±1.1)
**Female Age (x‐variable)**				
EF (%)	0.15	0.12	62.87	1.2 (±0.5)
EDVI (ml/m^2^)	−0.25	−0.31	84.45	−3.1 (±0.8)
ESVI (ml/m^2^)	−0.22	−0.17	30.18	−1.7 (±0.5)
SVI (ml/m^2^)	−0.17	−0.14	54.27	−1.4 (±0.6)
LVMI (g/m^2^)	−0.02	−0.02	49.91	−0.2 (±0.6)

A total of *n* = 782 volunteers satisfied the more stringent low‐risk factor inclusion criteria for the subset cohort, and the resulting data are presented in Appendices 1 (demographics) and 2 (MR parameters). There were no statistically significant differences between the means of any of the normalized LV variables when the data from the full cohort and the subset cohort were compared (*P* > 0.05 for all LV variables, including all comparisons when subdivided by gender and age).

**Figure 2:**
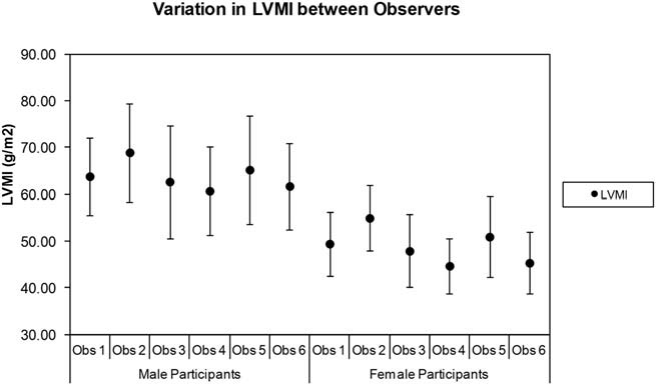
Variation in mean left ventricular mass index (LVMI; ± SD) between the different observers who participated in the data analysis. Of note is that observer 2 consistently derived the largest LVMI values and observer 4 consistently derived the smallest LVMI values. These data do not represent “true interobserver variation” since each study cohort was different for each observer. However, by using the LVMI and stratifying by gender, the component of the variation due to different cohort sizes and gender ratios has been minimized.

The variation in normalized mean LV measurements between the different segmentation observers is detailed in Table 4, and illustrated graphically in Fig. 2 for LVM (the most variable measurement). Although the group of volunteers segmented by each observer was different in each case, the data were stratified by age and gender, and normalized to BSA in order to make comparisons closely related to the segmentation technique itself. The consistency of the data between observers for mean EF data ranged from 66 ± 6% (observer 5) to 71 ± 5% (observer 1) for males, and from 68 ± 7% (observer 4) to 73 ± 5% (observer 1) for females. These were similar to the mean EF for the full cohort (Table 2) of 69 ± 6%. For the LV mass index, the consistency of the data ranged from 61 ± 9 g/m^2^ (observer 4), to 69 ± 11 g/m^2^ (observer 2) for males, and from 45 ± 6 g/m^2^ (observer 4) to 55 ± 7 g/m^2^ (observer 2) for females. These also compare favorably to the mean LV mass index for the entire cohort (Table 2) of 55 ± 12 g/m^2^.

Finally, a comparison of the 3.0T data with 1.5T data from elsewhere is presented in table 5. The mean EF was found to be marginally greater at 3.0T relative to 1.5T, but for all other variables the means were a little lower at 3.0T.

**Table 4:** LV Structure and Function Data (Mean ± SD) as Derived by Each of the Six Segmentation Observers

	No. Volunteers	Mean Age (yrs)	EF (%)	EDVI (ml/m^2^)	ESVI (ml/m^2^)	SVI (ml/m^2^)	LVMI (g/m^2^)
Males ‐ Obs 1	102 (6.7%)	55 ± 9	71 ± 5	74 ± 12	22 ± 6	52 ± 8	64 ± 8
Males ‐ Obs 2	106 (7.0%)	54 ± 8	68 ± 5	74 ± 13	24 ± 6	50 ± 9	69 ± 11
Males ‐ Obs 3	95 (6.3%)	53 ± 8	68 ± 5	79 ± 12	25 ± 6	54 ± 8	63 ± 12
Males ‐ Obs 4	90 (5.9%)	56 ± 8	67 ± 7	79 ± 15	27 ± 9	53 ± 10	61 ± 9
Males ‐ Obs 5	70 (4.6%)	55 ± 8	66 ± 6	78 ± 16	26 ± 8	52 ± 11	65 ± 12
Males ‐ Obs 6	111 (7.3%)	54 ± 8	67 ± 7	76 ± 12	26 ± 8	51 ± 8	62 ± 9
Females ‐ Obs 1	164 (10.8%)	54 ± 8	73 ± 5	64 ± 10	17 ± 5	46 ± 7	49 ± 7
Females ‐ Obs 2	145 (9.6%)	55 ± 9	70 ± 6	66 ± 10	20 ± 6	46 ± 7	55 ± 7
Females ‐ Obs 3	154 (10.2%)	55 ± 8	68 ± 6	70 ± 11	22 ± 6	47 ± 7	48 ± 8
Females ‐ Obs 4	161 (10.6%)	54 ± 8	69 ± 6	69 ± 11	22 ± 6	48 ± 7	45 ± 6
Females ‐ Obs 5	180 (11.9%)	54 ± 9	68 ± 7	70 ± 11	23 ± 8	47 ± 8	51 ± 9
Females ‐ Obs 6	137 (9.0%)	55 ± 8	68 ± 7	65 ± 10	21 ± 7	44 ± 6	45 ± 7

EF = ejection fraction, EDVI = end diastolic volume, ESVI = end systolic volume, SVI = stroke volume, LVMI = left ventricular mass.

**Table 5:** Comparison of Data Acquired at 1.5T (Taken From Ref. 11) With That Acquired in This Study

	LV	1.5T	1.5T	1.5T	3.0T	3.0T	3.0T	Difference
	Variable	Mean	SD	Range	Mean	SD	Range	1.5T ‐3.0T
MALES	EF (%)	67	5	57–77	68	6	55–80	−1
	EDV (ml)	160	27	106–214	155	28	100–210	5
	ESV (ml)	54	14	26–82	50	15	21–80	4
	SV (ml)	108	18	72–144	105	19	67–143	3
	LVM (g)	134	21	92–176	129	24	81–178	5
	EDVI (ml/m^2^)	81	12	57–105	77	13	50–103	4
	ESVI (ml/m ^2^)	26	6	14–38	25	7	10–40	1
	SVI (ml/m^2^)	54	6	42–66	52	9	34–70	2
	LVMI (g/m ^2^)	67	9	49–85	64	10	43–85	3
FEMALES	EF (%)	67	5	57–77	69	7	56–83	−2
	EDV (ml)	132	23	86–178	120	21	78–162	12
	ESV (ml)	44	11	22–66	37	12	13–61	7
	SV (ml)	87	15	57–117	82	14	54–111	5
	LVM (g)	98	21	56–140	87	17	54–120	11
	EDVI (ml/m^2^)	76	10	56–96	68	11	46–89	8
	ESVI (ml/m ^2^)	24	5	14–34	21	7	8–34	3
	SVI (ml/m^2^)	52	7	38–66	46	7	32–61	6
	LVMI (g/m ^2^)	61	10	41–81	49	8	33–65	12

Data are presented as the mean, SD, and range (defined as ± 2 SD of the mean). With the exception of EF, the calculated figures at 3.0T were all lower than those previously published at 1.5T and the difference was clearer in the female volunteer cohort.

## Discussion

In this study we present data describing MR LV structure and function in a large cohort of volunteers. The study methodology is similar to others performed previously.^1^, ^2^, ^3^ This work was prepared in response to the specific need for 3.0T MR data of this type as recently reported by Kawel‐Boehm et al.^11^ Statistical limitations associated with small study cohorts have been addressed by extending this work to include a large asymptomatic population, with full coverage across the adult age range to account for remodeling processes associated with the heart that occur with age.

In this study the means and ranges obtained for LV structure and function parameters at 3.0T are generally similar to those reported at 1.5T. The mean EF was marginally greater at 3.0T relative to 1.5T, but for all other variables the means were a little lower at 3.0T. The reason for these differences is not clear but may be related to variations in edge boundary perception due to changes in the *T*_1_ (and *T*_2_) relaxation times of the blood pool, myocardium, and other surrounding tissue structures. There may also be small differences in our study cohort demographics in relation to those reported from elsewhere. The greatest differences were noted for female volunteers, where the mean EDV and LVM parameters were 12 ml and 11 g lower, respectively, at 3.0T when compared to the previous pooled 1.5T data reported by Kawel‐Boehm et al.^11^ If the individual articles that contribute to this published range are scrutinized more closely, the best agreement to our 3.0T mean EDV (120 ml) is found in the work by Maciera et al,^3^ who obtained a mean EDV of 126 ml for their cohort of female volunteers in the 50–59 years age decade at 1.5T. Similarly, the closest agreement to our mean LVM (87 g) is reported in the work by Alfakih et al,^1^ who obtained a mean LVM of 88.1 g for their cohort of female volunteers in the 40–65 years age range at 1.5T. In other words, although some discrepancy exists between the pooled 1.5T data, there are individual contributions that agree quite closely with our findings at 3.0T.

When our 3.0T data were subdivided into male and female groups, the mean data for females were significantly lower than the means for males in all parameters studied, except for EF. This is expected and consistent with findings reported elsewhere.^2^ Alternative subdivision of the data into age decades (40s, 50s, 60s, and ≥70s) revealed that mean EF was virtually stable with age in males but rose a little with increasing age in females. Conversely, the mean LVMI was virtually stable with age in females but reduced with age in males. The other mean variables (EDVI, ESVI, and SVI) were found to reduce with age by varying amounts. These patterns of change over time are most similar to those reported by Hudsmith et al^2^ at 1.5T, where they compared cohorts of volunteers in groups with age stratification <35 years and >35 years. Similar patterns of change over time are also presented by Alfakih et al^1^ and Macieira et al^3^ at 1.5T.

The normalization of cardiac structure and function data is a complex area, and many approaches have been reported. Normalization to height,^25^ fat‐free mass (FFM),^26^ and weight^27^ have been proposed, but the method most commonly employed is normalization to BSA.^28^ The Mosteller index to BSA was chosen for this work since it is relatively simple, widely used, and validated on a wide range of subject sizes. Other normalization methods such as that proposed by DuBois and DuBois^29^ are also available, but this latter study was only validated on very small cohorts and is considered to be less meaningful at the lower and upper ranges of height and weight combinations.

In order to maintain optimal consistency, the study was conducted over a 5‐year period using the same scanner and the same RF coil combinations. No significant downtime was experienced over the duration of the work and the only minor change to the system over this period was an upgrade from software version VB15 to VB17, which did not noticeably affect the functionality of the MR protocols and analysis packages used. To this end, the experimental equipment was considered to remain stable for the duration of the experiment.

The data were acquired over such a large cohort that we elected to use multiple observers for the segmentation analysis. Each observer (six in total) was responsible for segmenting approximately *n* = 250 datasets. These data were stratified by gender and normalized to BSA (using the Mosteller formula) in order to account for body habitus variations between each of the cohorts allocated to each of the segmentation observers. This approach enables the variations in each LV parameter to be attributable to the segmentation technique, and not be influenced by the physical size or gender distribution of the cohort populations. The mean age of the cohorts allocated to each segmentation observer is also closely matched, which has ensured further consistency, ie, the segmentation technique itself is the dominant factor that forms the variation between observers. Although the segmentation technique was agreed beforehand and all observers were experienced, some real‐world differences between observers were apparent. The mean EF was slightly larger (and the ESVI slightly lower) for volunteers segmented by observer 1, suggesting that the observer was heavily excluding papillary muscles at end‐systole from the ESV blood pool volume. Similarly, the range of values for mean LVMI was variable in places, with observer 4 tending to generate slightly smaller mean values. While some of this variation may be due to the fact that different volunteers were included in each cohort (an accepted limitation of the study design), the likelihood is that it is mostly down to subtle variations in the segmentation technique between the observers (similar variability figures are reported in studies by Chuang et al^30^ and Suinesiaputra et al^31^). It was, however, most encouraging to note that the generated data were generally very similar between all of the observers.

The use of a 3.0T scanner for this work was proposed on the basis that the theoretical improvement to the signal‐to‐noise ratio might be traded for faster scanning (ie, more LV SA slices per breath‐hold) and therefore faster volunteer throughput. However, in reality much of the “time saved” (relative to 1.5T) was required for the process of image optimization, eg, the use of optimized volunteer‐specific shimming techniques (eg, “frequency scout”) and targeted shim regions placed over the area of interest during the examination in order to eradicate resonant‐offset banding and flow‐related artifacts. Although relatively little “time‐saving” was achieved, the comparison with existing 1.5T data should help to provide support data for future population‐based research studies that may utilize scanners at both field strengths.

Limitations of the study include the fact that the software used was not easily able to account for papillary muscle volumes, leading to possible small variation between observers as to how the papillary structures would in practice be treated. A further limitation of the study is that no direct equivalent 1.5T data were available and no spoiled gradient echo data were acquired for comparative purposes. The size of the study was such that this was considered prohibitive in terms of time, but similar comparisons are available in the existing literature for 1.5T data^12^ and it is likely that similar trends would be seen at 3.0T. Finally, it is accepted that the inclusion criteria used to identify certain risk factors associated with CVD during the recruitment phase were a little more relaxed than current recommendations. The decision to select asymptomatic volunteers with a BNP greater than the gender‐specific median was made with a view to undertaking further MR examinations on the same volunteer cohort as they become older. However, the risk of this significantly confounding the results of this study is minimal: we have knowingly excluded anybody with clinically apparent CVD in this work, and in addition to which there were no statistical differences detected within any of the cardiac MR parameters when only those with a BNP <2 SD above the gender‐specific mean (and therefore associated with normal limits) were included in the analysis.

In conclusion, we describe LV reference ranges in a population‐based MR study of volunteers asymptomatic of CVD at 3.0T. The resulting figures are similar to those normal ranges previously reported at 1.5T, and changes with age and gender also follow similar patterns. Data acquired from the full cohort are very similar to those derived from a subgroup with lower risk factors for CVD, suggesting that CVD risk at these levels does not contribute a significant effect. These baseline data might also enable future monitoring of LV changes over time as/when individuals within the cohort require future MR examinations.

## Conflict of Interest

The authors declare that they have no competing interests.

**Table Appendix 1:** Demographic information related to anatomical size for a subset of volunteers (*n* = 782) with lower risk factors for CVD, based on 1) BP <140/90 mmHg, 2) nonsmokers, and 3) BNP <2 SD above the original cohort gender‐specific mean.

ABSOLUTE	No Volunteers	Height (m)	Weight (kg)	BMI (kg/m^2^)	BSA (Mosteller)	BSA (DuBois)
All	782	1.68 (0.10)	74.99 (14.30)	26.09 (4.23)	1.87 (0.21)	1.85 (0.20)
Males	299	1.78 (0.07)	83.78 (11.26)	26.47 (3.21)	2.03 (0.16)	2.02 (0.15)
Females	483	1.64 (0.07)	69.30 (13.13)	25.85 (4.74)	1.77 (0.18)	1.75 (0.16)
						
Males (40s)	118	1.79 (0.07)	85.37 (11.24)	26.60 (3.35)	2.06 (0.15)	2.04 (0.15)
Males (50s)	135	1.77 (0.07)	83.64 (11.51)	26.64 (3.09)	2.02 (0.16)	2.01 (0.16)
Males (60s)	38	1.77 (0.07)	79.45 (10.09)	25.38 (3.12)	1.97 (0.14)	1.96 (0.13)
Males (≥70s)	8	1.76 (0.04)	83.08 (8.20)	26.89 (3.01)	2.01 (0.10)	1.99 (0.09)
						
Females (40s)	176	1.65 (0.06)	70.68 (13.78)	26.14 (4.84)	1.79 (0.19)	1.77 (0.17)
Females (50s)	185	1.64 (0.07)	70.36 (13.74)	26.24 (5.09)	1.78 (0.18)	1.76 (0.16)
Females (60s)	107	1.63 (0.07)	65.90 (10.53)	24.89 (3.90)	1.72 (0.15)	1.71 (0.14)
Females (≥70s)	15	1.62 (0.07)	64.43 (9.75)	24.70 (3.89)	1.70 (0.15)	1.68 (0.14)

**Table Appendix 2:** LV structure and function data acquired in the *n* = 782 subset of volunteers. When compared with the equivalent normalized data acquired from the whole cohort (Table 2), there were no significant differences between the means of any variable (*P* > 0.05 for all data, including subcomparisons stratified by age and gender).

ABSOLUTE	No Volunteers	EF (%)	EDV (ml)	ESV (ml)	SV (ml)	LVM (g)
All	782	69 ± 6	134 ± 29	43 ± 14	92 ± 19	102 ± 28
Males	299	67 ± 6	158 ± 25	52 ± 14	106 ± 18	128 ± 22
Females	483	70 ± 6	119 ± 20	37 ± 11	83 ± 14	86 ± 16
						
Males (40s)	118	66 ± 6	165 ± 26	56 ± 13	109 ± 20	133 ± 24
Males (50s)	135	68 ± 6	154 ± 24	49 ± 14	104 ± 17	126 ± 20
Males (60s)	38	68 ± 7	153 ± 24	50 ± 17	103 ± 14	123 ± 19
Males (≥70s)	8	67 ± 4	154 ± 12	51 ± 10	102 ± 7	121 ± 13
						
Females (40s)	176	68 ± 5	125 ± 20	40 ± 11	85 ± 13	86 ± 15
Females (50s)	185	70 ± 6	121 ± 20	37 ± 10	84 ± 14	87 ± 17
Females (60s)	107	71 ± 6	109 ± 15	31 ± 9	78 ± 12	84 ± 14
Females (≥70s)	15	73 ± 5	105 ± 21	29 ± 9	76 ± 13	81 ± 14

**NORMALISED**	No Volunteers	EF (%)	EDVI (ml/m^2^)	ESVI (ml/m^2^)	SVI (ml/m^2^)	LVMI (g/m^2^)
All	782	69 ± 6	72 ± 12	23 ± 7	49 ± 8	54 ± 11
Males	299	67 ± 6	78 ± 12	26 ± 7	52 ± 8	63 ± 10
Females	483	70 ± 6	68 ± 10	21 ± 6	47 ± 7	49 ± 8
						
Males (40s)	118	66 ± 6	80 ± 12	27 ± 7	53 ± 9	65 ± 11
Males (50s)	135	68 ± 6	76 ± 12	25 ± 7	52 ± 8	63 ± 9
Males (60s)	38	68 ± 7	78 ± 12	26 ± 9	52 ± 7	62 ± 10
Males (≥70s)	8	67 ± 4	76 ± 6	26 ± 5	51 ± 3	60 ± 7
						
Females (40s)	176	68 ± 5	70 ± 10	23 ± 6	48 ± 7	48 ± 7
Females (50s)	185	70 ± 6	68 ± 11	21 ± 6	47 ± 8	49 ± 8
Females (60s)	107	71 ± 6	63 ± 8	18 ± 5	45 ± 7	49 ± 7
Females (≥70s)	15	73 ± 5	62 ± 10	17 ± 5	45 ± 7	48 ± 8
